# *Brucella*-Induced Impairment of Decidualization and Its Impact on Trophoblast Function and Inflammatory Profile

**DOI:** 10.3390/ijms26178189

**Published:** 2025-08-23

**Authors:** Lucía Zavattieri, Rosario Macchi, Andrea Mercedes Canellada, Matías Arturo Pibuel, Daniela Poodts, Mariana Cristina Ferrero, Pablo Cesar Baldi

**Affiliations:** 1 Cátedra de Inmunología, Facultad de Farmacia y Bioquímica, Universidad de Buenos Aires, Buenos Aires 1113, Argentina; mv.lzavattieri@gmail.com (L.Z.); rochimacchi2@gmail.com (R.M.); acanell@ffyb.uba.ar (A.M.C.); pibuelmatias@gmail.com (M.A.P.); dani.poodts@gmail.com (D.P.); 2 Instituto de Estudios de la Inmunidad Humoral (IDEHU), CONICET—Universidad de Buenos Aires, Buenos Aires 1033, Argentina

**Keywords:** endometrial stromal cells, decidua, trophoblasts, *Brucella*, chemokines, pregnancy complications, infertility

## Abstract

*Brucella* infection is associated with an increased risk of adverse obstetric outcomes in humans and animals. Decidualization, a process involving structural and functional changes in endometrial stromal cells, is essential for proper trophoblast implantation and placental development. Trophoblasts’ migration and their ability to invade the decidua and to undergo tubulogenesis, critical for proper implantation and placental development, are normally promoted by decidual cells. We evaluated whether *Brucella* infection of human endometrial stromal cells (T-HESC cell line) affects their ability to decidualize and to promote trophoblast functions. Infection of T-HESC cells with either *B. abortus*, *B. suis*, or *B. melitensis* resulted in deficient decidualization (as revealed by reduced prolactin levels) and an increased production of proinflammatory chemokines (C-X-C motif chemokine ligand 8 -CXCL8- and C-C motif chemokine ligand 2 -CCL2-) as compared to uninfected cells subjected to decidualization stimuli. In addition, conditioned media (CM) from infected decidualized T-HESC induced an inflammatory response (CXCL8, CCL2 and interleukin-6 -IL-6) in human trophoblasts (Swan-71 cell line) but reduced their ability to produce progesterone. Trophoblasts preincubated with this CM also had reduced migration, invasion, and tubulogenesis capacities, and this impairment was mediated, at least in part, by CXCL8 and CCL2. Moreover, infection of decidual stromal cells impaired the adhesion and spreading of blastocyst-like spheroids formed by Swan-71 cells. *Brucella* infection also affected the chemotactic capacity of decidual stromal cells for trophoblasts. Overall, these results suggest that *Brucella* infection of endometrial stromal cells impairs key processes required for successful implantation and placental development.

## 1. Introduction

Brucellosis is a globally distributed zoonotic disease caused by Gram-negative bacteria of the *Brucella* genus. The disease primarily affects domestic and wild animals, which act as reservoirs, facilitating transmission to humans [1]. Among the species most pathogenic to humans are *B. melitensis*, *B. suis*, and *B. abortus*. The infection may be systemic or may be localized, affecting preferentially a given organ. The most frequently described signs and symptoms of human brucellosis include fever, asthenia, myalgia, arthralgia, sweats, lymphadenopathy, hepatomegaly, and splenomegaly. The disease has a tendency to chronicity and persistence, becoming a granulomatous disease capable of affecting different organs. Among localized forms of the disease, those affecting bones and joints (arthritis, sacroiliitis, spondylitis) are the most prevalent, followed by neurobrucellosis (meningitis, encephalitis, etc.), orchiepididymitis, and hepatic involvement [2,3,4,5,6]. Severe vascular complications, including infective endocarditis and mycotic aneurysms, are less frequent but may be associated with increased risk of death [7].

In animals, brucellosis is closely linked to reproductive disorders, including abortion, premature birth, orchitis, epididymitis, and infertility. In pregnant women, numerous studies have reported an increased risk of adverse obstetric outcomes such as preterm delivery, spontaneous abortion, fetal death, and low birthweight [8,9,10,11,12,13]. According to different reports the rate of spontaneous miscarriage related to human brucellosis ranges from 18.6 to 73.3% [14,15] depending on the infecting species, the infection route, and the median age of the mothers. Most cases occur during the first and second trimesters of gestation. Whereas abortion has been related to the three main *Brucella* species (*B. melitensis*, *B. suis*; *B. abortus*), there is an increased frequency for *B. melitensis*, which may relate to its heightened virulence or a high consumption of fresh goat milk products in endemic areas. Notably, infection can be transmitted to the fetus, primarily through transplacental spread, with cases reported for *B. abortus* and *B. melitensis* [16,17]. Given the association between *Brucella* infection and reproductive complications, understanding its impact on key processes at the maternal-fetal interface is crucial.

Establishing a successful pregnancy relies on maternal endometrial receptivity during the implantation window. A pivotal event in this process is decidualization, which involves structural and functional changes in endometrial stromal cells, including cell enlargement, spiral artery remodeling, and immune cell infiltration by macrophages and uterine natural killer cells [18]. Endometrial stromal cells, the predominant cell type in the endometrium, undergo cyclic differentiation during the luteal phase in response to rising levels of estrogen and progesterone [19]. This differentiation transforms stromal cells from a fibroblast-like morphology into epithelioid-like decidual cells, a process that in humans occurs even in the absence of implantation and reaches full maturity at the onset of gestation. Decidualization not only supports implantation by promoting the secretion of key factors such as prolactin but also facilitates immune tolerance by attracting predominantly anti-inflammatory leukocytes, preventing excessive recruitment of cytotoxic T cells [20]. Additionally, decidual cells secrete chemokines that promote trophoblast invasion and angiogenesis, essential for early pregnancy development [21].

At the maternal–fetal interface, the decidua and the placenta form a highly specialized structure critical for pregnancy maintenance. The placenta contains three types of trophoblasts: cytotrophoblasts, which are mononuclear proliferative cells; syncytiotrophoblasts, which form a multinucleated layer covering the chorionic villi; and extravillous trophoblasts (EVTs). Among these, EVTs invade the decidua to anchor the placenta and remodel maternal spiral arteries, ensuring adequate blood flow to the fetus. Together, the decidua and trophoblasts contribute to the production of essential hormones and nutrients required for fetal growth. Proper decidualization and trophoblast invasion are, therefore, fundamental processes that, if disrupted, may lead to pregnancy complications.

Although the uterus provides a unique immune environment that supports the development of the semi-allogeneic fetus, it remains susceptible to infections. Pathogens may access the endometrium via ascending routes from the vagina or through hematogenous circulation [22], potentially altering its function and triggering inflammatory responses that can compromise pregnancy [23]. Infections have been implicated in up to 15% of early pregnancy losses [24,25]. Decidual and non-decidual stromal cells express various pathogen recognition receptors, including toll-like receptors (TLRs) and nod-like receptors (NLRs), which detect microbial components and initiate immune responses through the secretion of inflammatory cytokines, chemokines, and metalloproteinases [26,27]. This innate immune defense is essential for limiting infection but can also lead to tissue damage and impaired decidual function when dysregulated.

Altered decidualization, measured by reduced expression of decidual markers such as prolactin (PRL), has been linked to pregnancy complications, including miscarriage, preeclampsia, and intrauterine growth restriction [28,29,30]. Moreover, infections by bacteria such as *Chlamydia trachomatis* have been shown to impair decidualization and decidual function in uterine stromal cells [31]. Despite these findings, little is known about how bacterial infections, such as those caused by *Brucella*, affect the interaction between endometrial stromal cells and EVTs at the maternal–fetal interface.

We previously demonstrated that *B. abortus* infects fully decidualized and non-decidualized endometrial stromal cells, inducing a proinflammatory response [32]. Moreover, a recent study in mice showed that lipopolysaccharide from *B. suis* S2 disrupts decidualization and reduces implantation rates [33]. In this study, we aimed to investigate the effects of infection with highly virulent *Brucella* species on stromal cell decidualization, as well as its subsequent effects on trophoblast migration, adhesion, invasion, and immune responses in the context of trophoblast–decidua interactions.

## 2. Results

### 2.1. Brucella spp. Infection of Human Endometrial Stromal Cells Results in Deficient Decidualization

Infertility is linked to *Brucella* infections. Even when there is no pregnancy, *Brucella* can colonize the uterus like other hematogenously disseminated infections [34,35,36]. Therefore, we evaluated whether infection with virulent *Brucella* species affects the subsequent decidualization process of uterine endometrial stromal cells.

**Figure 1:**
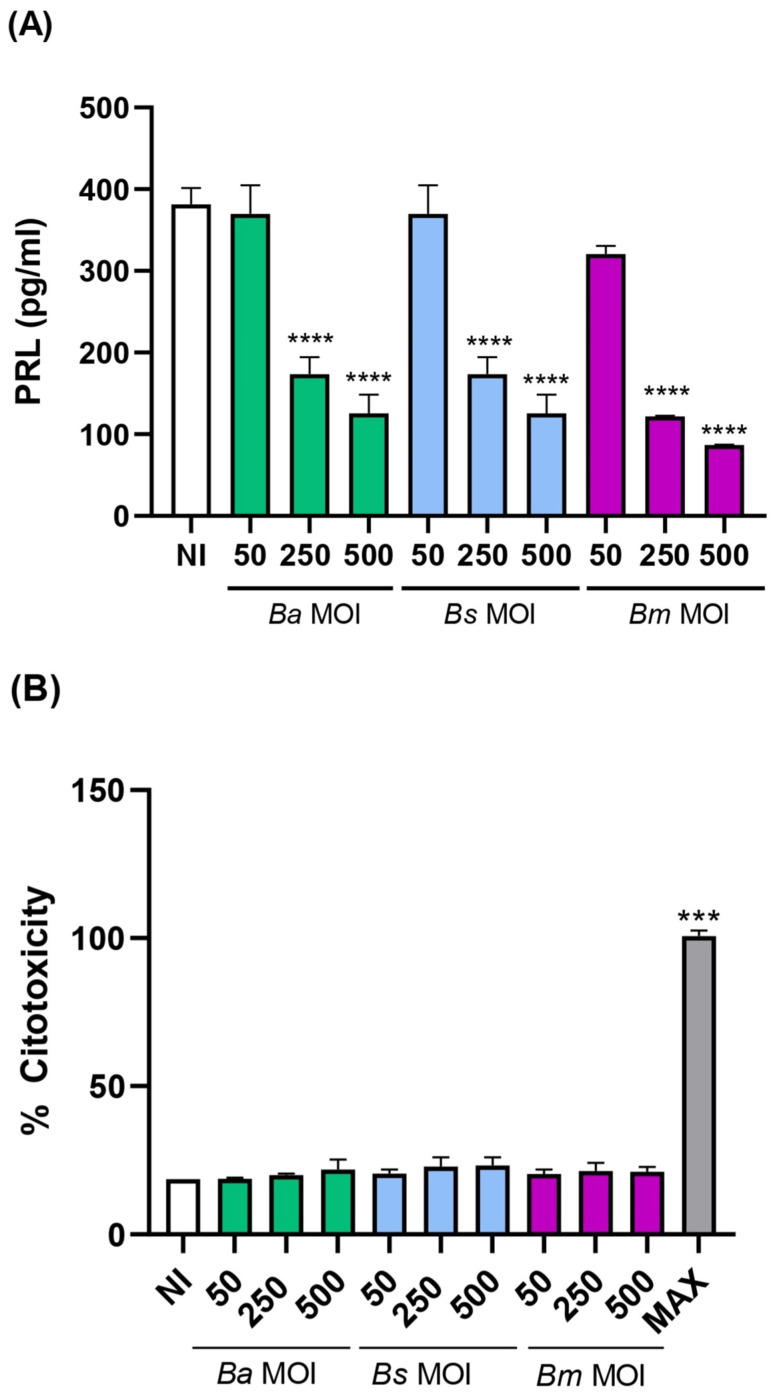
*Brucella* preinfection impairs the decidualization of human endometrial stromal cells. Endometrial stromal cells from the T-HESC line were infected with *B. abortus* (*Ba*), *B. melitensis* (*Bm*), or *B. suis* (*Bs*) at different multiplicities of infection (MOI) or were left uninfected (NI) as controls. At 24 h post-infection, both infected and non-infected cells were subjected to the decidualization protocol. Culture supernatants were collected at day 6 post-decidualization, and prolactin (PRL) levels were quantified by ELISA (**A**). In parallel, lactate dehydrogenase release was measured in culture supernatants to assess cytotoxicity using a commercial non-radioactive cytotoxicity assay (**B**). Cytotoxicity was expressed as percentage of cell lysis, with the 100% control (Max) obtained by hypotonic lysis of an equal number of non-infected cells. Results are expressed as mean ± SD of three independent experiments performed in duplicate. *** *p*< 0.001, **** *p* < 0.0001 versus NI.

As shown in Figure 1A, prior infection with *Brucella* significantly reduced PRL production in a multiplicity of infection (MOI)-dependent manner at 6 days post-decidualization compared to non-infected cells. No changes in PRL secretion were observed at earlier time points (Figure S1). These differences were not attributable to a cytotoxic effect of the infection, as lactate dehydrogenase (LDH) release measured in culture supernatants did not show significant differences between infected and control cells (Figure 1B). These findings indicate that prior *Brucella* spp. infection impairs the ability of endometrial stromal cells to undergo proper decidualization, which could contribute to the implantation and pregnancy maintenance disorders associated with *Brucella* infections.

### 2.2. Brucella spp. Infection of Human Endometrial Stromal Cells Induces a Proinflammatory Chemokine Response

We previously demonstrated that *B. abortus* can induce the production of the inflammatory chemokines C-X-C motif chemokine ligand 8 (CXCL8, neutrophil chemoattractant) and C-C motif chemokine ligand 2 (CCL2, monocyte chemoattractant) in both fully decidualized and non-decidualized stromal cells, which may contribute to the inflammation-driven gestational complications of brucellosis [32]. To further investigate the impact of prior *Brucella* infection on the production of inflammatory factors by decidualized T-HESC cells, CXCL8, interleukin-6 (lL-6) and CCL2 levels were measured in culture supernatants.

**Figure 2:**
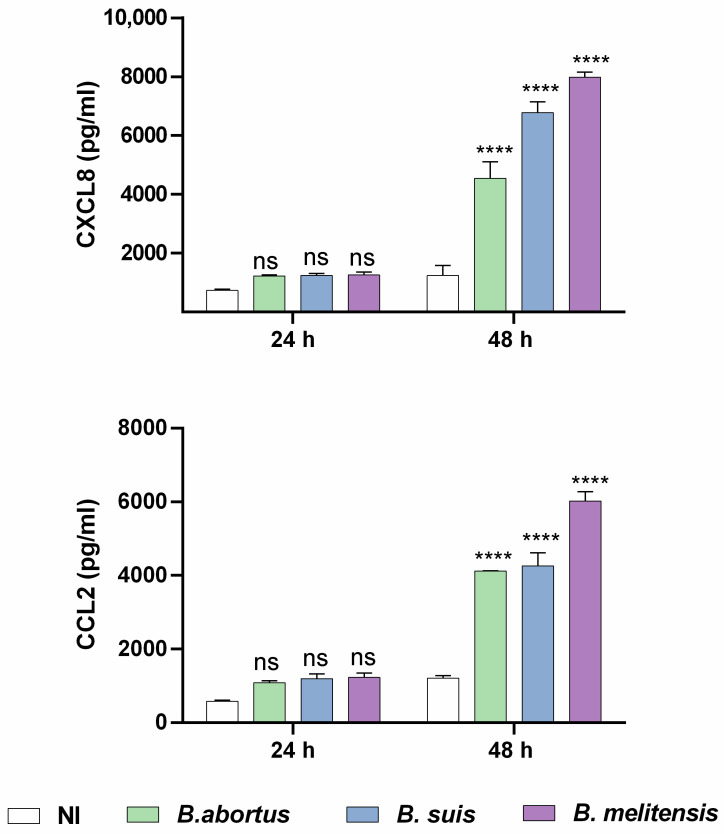
*Brucella* preinfection induces a proinflammatory chemokine response in decidualized human endometrial cells. T-HESC cells were infected with the different *Brucella* species (MOI 250) or were left uninfected (NI) as controls. At 24 h post-infection, both infected and non-infected cells were subjected to the decidualization protocol for 8 days. Cells were incubated for a further 24 or 48 h, and at culture supernatants were harvested for measuring CXCL8 and CCL2 using commercial ELISA kits. Results are expressed as mean ± SD of three independent experiments performed in duplicate. ns: non-significant; **** *p* < 0.0001 versus NI at the corresponding time point.

The results show that prior *B. abortus*, *B. melitensis*, and *B. suis* infection significantly increased the secretion of both CXCL8 and CCL2 compared to non-infected but decidualized cells (Figure 2). In contrast, infection with all tested *Brucella* species did not increase IL-6 production. Taken together, these findings indicate that infection with virulent *Brucella* strains alters the secretome of decidualized stromal cells, affecting not only prolactin production but also enhancing the secretion of proinflammatory chemokines. This dysregulation may compromise trophoblast functionality and, consequently, impair placental development.

### 2.3. Factors Released by Infected Decidualized Stromal Cells Induce an Inflammatory Response in Trophoblasts

The maintenance of a regulated inflammatory microenvironment during the gestational period is crucial to ensure a successful pregnancy. Whereas decidualized stromal cells support pregnancy through their interactions with trophoblasts, in the context of infection they may contribute to an altered secretome that affects trophoblast functionality and inflammatory profile. To investigate this, we analyzed the effects of conditioned medium (CM) from *B. abortus*-infected and later decidualized stromal cells (CM *Ba*) on the immune response of trophoblasts from the Swan-71 cell line. These cells were stimulated with CM *Ba* or CM from non-infected stromal cells (CM NI) for 24 or 48 h, and CXCL8, IL-6, and CCL2 levels were quantified by ELISA. Cytokine levels present in the CMs were subtracted from those detected in the supernatants of stimulated trophoblasts to account for baseline cytokine presence.

**Figure 3:**
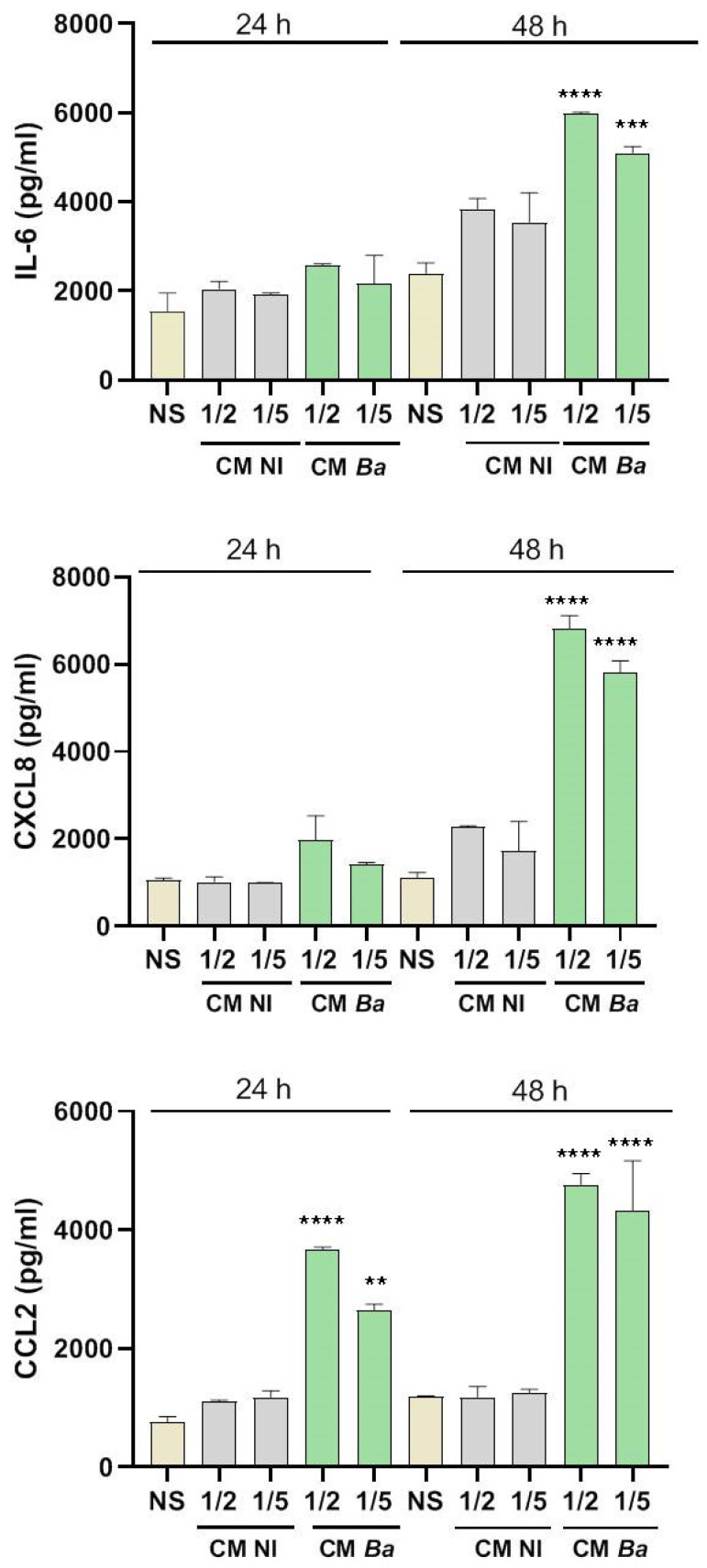
Factors secreted by *Brucella*-infected decidualized stromal cells stimulate a proinflammatory response in trophoblasts. Trophoblasts from the Swan-71 cell line were stimulated for 24 or 48 h with conditioned medium from *B. abortus*-infected and later decidualized THESC cells (CM *Ba*) or from non-infected but decidualized cells (CM NI). After stimulation, CXCL8, IL-6, and CCL2 levels in the culture supernatants were quantified by ELISA. Cytokine levels present in the CMs were subtracted from those detected in the supernatants of stimulated trophoblast to account for baseline cytokine presence. Results are expressed as mean ± SD of three independent experiments performed in duplicate. NS: non-stimulated control. ** *p* < 0.01; *** *p* < 0.001; **** *p* < 0.0001 versus NS.

Our results indicate that exposure to CM from *Brucella*-infected decidual cells significantly increased the production of IL-6 and CXCL8 in trophoblasts compared to non-stimulated controls (Basal) at 48 h post-stimulation (Figure 3). Additionally, CCL2 secretion was elevated at both 24 and 48 h post-stimulation (Figure 3, lower panel). In contrast, trophoblasts stimulated with CM NI did not show increased cytokine or chemokine production compared to the basal control, suggesting that the inflammatory response of trophoblasts is specifically triggered by infection-induced changes in the decidual secretome. Overall, these results highlight a potential mechanism by which infected maternal tissues influence placental immunity.

### 2.4. Brucella Infection Reduces the Ability of Decidualized T-HESC Cells to Stimulate Progesterone Production in Trophoblasts

Progesterone has a crucial role in pregnancy maintenance through modulation of maternal immune responses, reduction of uterine contractility, improvement of utero–placental circulation and promotion of extravillous trophoblasts invasion to the decidua [37]. Progesterone also stimulates the proliferation and differentiation of endometrial stromal cells [19]. Given the extensive cross talk between trophoblasts and decidual cells during pregnancy [38,39], and that some factors produced by decidual cells can stimulate progesterone secretion in trophoblasts [40], we evaluated whether CM from T-HESC decidual cells may induce progesterone production in Swan-71 trophoblasts and whether this effect may be altered by *Brucella* infection.

**Figure 4:**
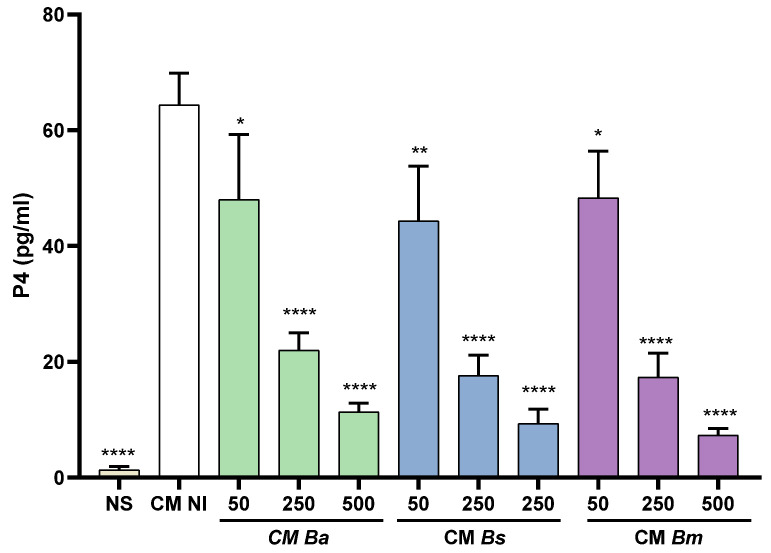
*Brucella* infection reduces the ability of decidualized T-HESC cells to stimulate progesterone (P4) production in trophoblasts. Swan-71 trophoblasts were stimulated with CM from *B. abortus*-, *B. suis*-, or *B. melitensis*-infected and later decidualized THESC cells (CM *Ba*, CM *Bs*, and CM *Bm*, respectively) or from non-infected but decidualized cells (CM NI), or were kept non-stimulated (NS). At 48 h post-stimulation, culture supernatants were harvested to measure progesterone with a commercial ELISA. The results are expressed as mean ± SD of three independent experiments performed in duplicate. * *p* < 0.05; ** *p* = 0.01; **** *p* = 0.0001 versus CM NI.

Trophoblasts were stimulated or not (basal) with CM *Ba* or CM from *B. suis* and *B. melitensis*-infected and later decidualized T-HESC cells (CM *Bs* and CM *Bm*) or CM NI. Progesterone levels were measured 48 h later in the supernatants of the stimulated cultures. As shown in Figure 4, trophoblasts stimulated with CM NI significantly increased their secretion of progesterone as compared to the basal control. In contrast, progesterone levels induced by stimulation with CM from decidualized T-HESC previously infected with any of the *Brucella* species tested were significantly lower and this reduction was proportional to the MOI used for infection. These results suggest that *Brucella* infection may induce changes in the decidual soluble factors involved in progesterone secretion by trophoblasts.

### 2.5. Brucella Infection of Decidualized Stromal Cells Impairs Outgrowth of Trophoblast Spheroid in Co-Culture Models

Proper implantation requires dynamic interactions between invading trophoblast cells and receptive decidual stromal cells, which undergo extensive remodeling during early pregnancy. Decidualization promotes a supportive environment for embryo implantation [41]. Given that *Brucella* can infect and alter stromal cell function, we hypothesized that infection of decidual stromal cells may impair their interaction with trophoblasts, thereby disrupting blastocyst-like spheroid adhesion (BLS) and spreading (a surrogate of impaired implantation).

**Figure 5:**
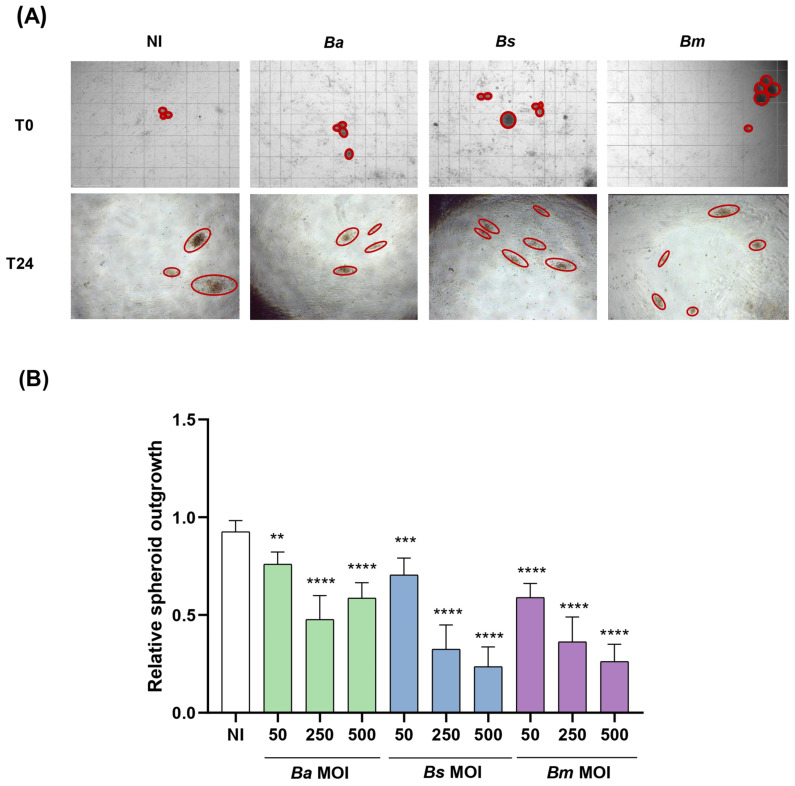
*Brucella* infection of decidual stromal cells impairs the adhesion of trophoblast spheroids. THESC cells were infected with *B. abortus*, *B. suis*, or *B. melitensis* at different multiplicities of infection (*Ba* MOI, *Bs* MOI, and *Bm* MOI) or were left uninfected (NI), and were then decidualized. Blastocyst-like spheroids formed by non-infected trophoblasts were then laid on decidualized cells. Pictures were taken at 0 h and 24 h to measure the area of spheroids outgrowth. Representative images at 40× magnification showing the area of spheroids outgrowth (red circles) on THESC infected with *Ba*, *Bs*, or *Bm* at an MOI of 500 (**A**). Relative spheroid outgrowth area expressed as the mean ± SD of two independent experiments, each performed in duplicate (**B**). Asterisks over bars indicate differences versus the NI condition (** *p* < 0.01; *** *p* < 0.001; **** *p* < 0.0001).

As shown in Figure 5, previous infection of stromal cells with any of the *Brucella* species tested reduced the spreading of spheroids over decidual monolayers in an MOI-dependent manner, compared to the uninfected control, as indicated by a significant decrease in BLS area. These findings suggest that *Brucella* infection of decidual stromal cells negatively affects trophoblasts’ adhesion to the decidua, potentially disrupting the decidua–trophoblast interaction required for proper implantation and placental development.

### 2.6. Conditioned Medium from Infected Decidualized Stromal Cells Affects the Migration of Trophoblast

Trophoblast migration is a critical process for proper placental development and remodeling of the maternal–fetal interface. Disruptions in this process can contribute to pregnancy complications. Taking into account that decidual infection by *Brucella* reduced the spreading of BLS over decidual monolayers, and that the CMs of infected decidualized stromal cells modulated trophoblast immune responses, we next evaluated, using a wound healing assay, whether trophoblast migration, a key process for BLS spreading, may be also influenced by these CMs.

**Figure 6:**
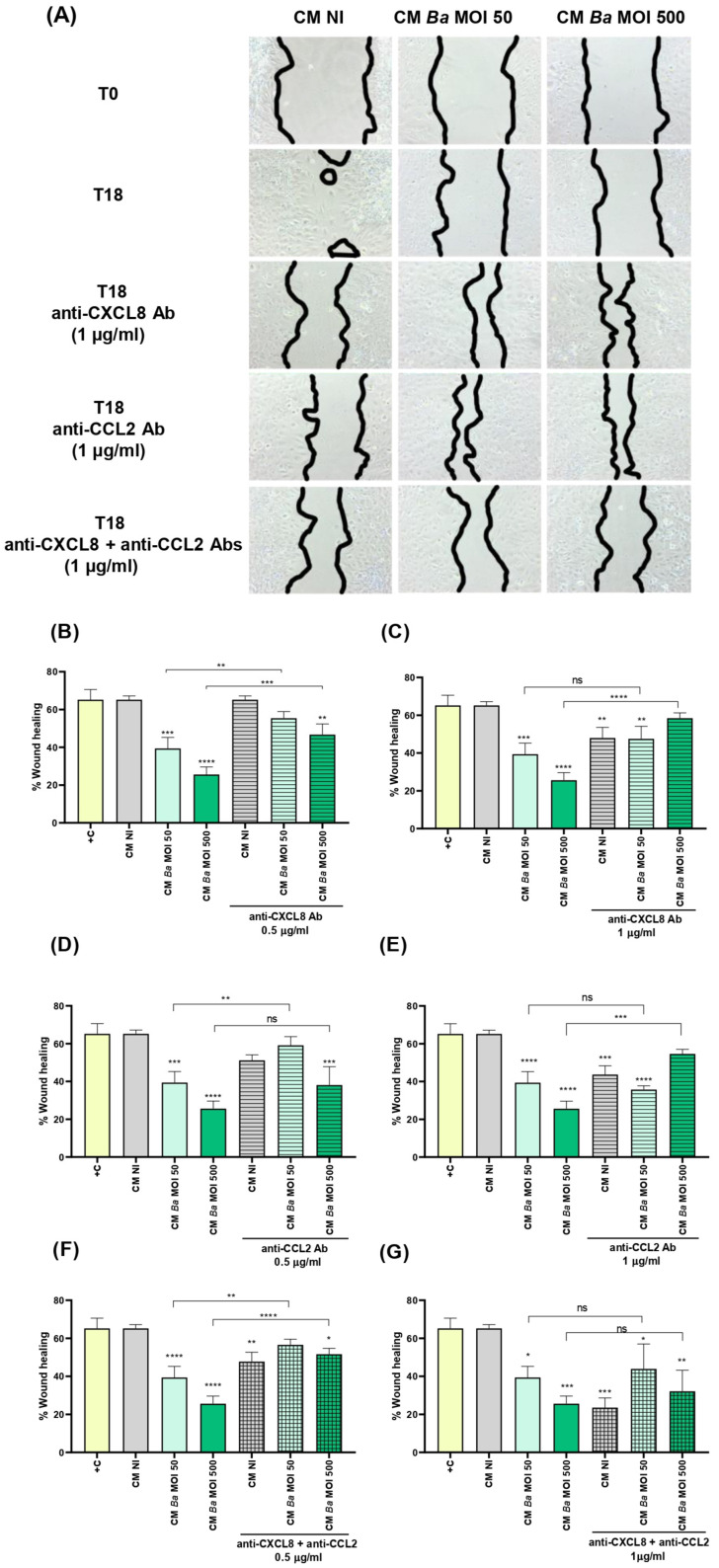
Conditioned medium from *Brucella abortus*-infected decidual cells impairs trophoblast migration. Swan-71 trophoblasts were dispensed at 5 × 10^4^ cells/well and were grown until confluence. A scratch was performed in the culture with a pipette tip, and then cells were stimulated with conditioned medium from *B. abortus*-infected and later decidualized THESC cells (CM *Ba*) or from non-infected but decidualized cells (CM NI). Stimulation with the corresponding CM was maintained during the whole assay. Swan-71 cells cultured in DMEM/F-12 supplemented with 10% FBS were used as a positive control of migration (+C). Wound closure was evaluated by taking images at 0 h (T0) and 18 h (T18) post-stimulation from the same microscopic field. Panel A shows representative 40× magnification images of wound healing in Swan-71 cells treated with CM-Ba or CM-NI at T0 and T18. Images were processed using ImageJ software (version 1.54p). The percentage of wound healing was calculated as: [(area time 0 h − area time = 18 h)/area time = 0 h] × 100. In parallel experiments, CM *Ba* and CM NI were preincubated (or not) for 1 h with two concentrations (0.5 and 1 µg/mL) of neutralizing antibodies against CXCL8 (**A**–**C**) or CCL2 (**A**,**D**,**E**) or a mixture of both (**A**,**F**,**G**) before performing the wound healing assay. Results are expressed as mean ± SD of three independent experiments performed in duplicate. Asterisks over bars indicate differences versus the +C condition, whereas asterisks over lines indicate differences between antibody-treated and untreated conditions (* *p* < 0.05; ** *p* < 0.01; *** *p* < 0.001; **** *p* < 0.0001; ns: non-significant).

To this end, a wound was created on monolayers of Swan-71 cells, which were subsequently stimulated for 18 h with either CM *Ba*, CM *Bs*, CM *Bm*, or CM NI. Images of the wound were taken at 0 h post-stimulation and at the end of the assay. As shown in Figure 6, stimulation with CM *Ba* obtained at two different MOI significantly inhibited trophoblast wound closure compared to stimulation with CM NI. No significant differences in wound closure were detected between cells stimulated with CM NI and those treated with culture medium supplemented with 10% fetal bovine serum (FBS, positive control). A similar inhibition was observed for CM *Bs* and CM *Bm*, as depicted in Figures S2 and S3, respectively.

To assess whether the chemokines CXCL8 and CCL2, induced by *Brucella* spp. infection and present in the CM *Ba*, CM *Bs*, and CM *Bm*, might be involved in wound closure inhibition, we preincubated CM from infected cells and CM NI for 1 h with two concentrations of neutralizing antibodies against CXCL8 and CCL2 (0.5 and 1 µg/mL) before repeating the wound healing assay. As shown in Figure 6A, neutralization of CXCL8 with the lower antibody concentration (0.5 µg/mL) fully restored the migration of CM *Ba* MOI 50, with no significant differences compared to the untreated control (CM NI). However, in CM *Ba* MOI 500, low-dose CXCL8 neutralization only partially restored migration, as significant differences remained compared to the untreated CM *Ba* MOI 500. Notably, high-dose CXCL8 neutralization (1 µg/mL) completely reversed the migration inhibition induced by CM *Ba* MOI 500, restoring it to untreated CM NI levels (Figure 6B). Interestingly, high-dose CXCL8 neutralization also partially reduced trophoblast migration in both CM NI and CM *Ba* MOI 50, compared to untreated CM NI (Figure 6B).

Similar to the effects observed with CXCL8 neutralization, blocking CCL2 in CM *Ba* MOI 50 at the lower tested antibody dose restored migration, achieving wound closure levels comparable to CM NI (Figure 6C). Although no significant differences were observed in wound closure between CM *Ba* MOI 500 treated with the lower dose of neutralizing anti-CCL2, high-dose CCL2 neutralization further enhanced CM-induced migration (Figure 6D). Consistent with the findings for CXCL8, treatment with neutralizing anti-CCL2 at 1 µg/mL significantly reduced migration in both CM NI and CM *Ba* MOI 50 compared to untreated CM NI. Furthermore, pre-treatment of both CM *Ba* MOI 50 and MOI 500 with low-dose neutralizing antibodies against CXCL8 and CCL2 restored wound closure to levels comparable to untreated CM NI (Figure 6E). In contrast, treatment with both neutralizing antibodies at the higher dose not only failed to improve wound closure in CM *Ba* MOI 50 and MOI 500 but also reduced CM NI-induced wound closure compared to untreated CM NI (Figure 6F).

Similar results were observed in the trophoblast wound closure induced by CM Bs following neutralization of CXCL8, CCL2, or both (Figure S2). Regarding *B. melitensis* infections, in contrast to what was observed for CM from THESC infected with *B. abortus* and *B. suis*, treatment with the lower doses of neutralizing antibodies against CXCL8 or CCL2 failed to reverse the inhibition of wound closure induced by CM *Bm* MOI 50 and MOI 500 (Figure S3). Only the highest dose of individual neutralizing antibodies could counteract the inhibitory effect on wound closure induced by CM *Bm* MOI 500. However, this dose of neutralizing antibody also inhibited migration induced by CM NI, compared to the respective untreated condition (Figure S3). Notably, pretreatment of CM *Bm* with both neutralizing antibodies at the lowest dose, but not at the highest dose, fully reversed the wound closure inhibition induced by CM *Bm* MOI 50 and MOI 500, compared to CM NI. In addition, high doses of both neutralizing antibodies strongly inhibited wound closure induced by CM NI.

Overall, these findings suggest that a delicate balance between CXCL8 and CCL2 levels regulates trophoblast migration, either promoting or inhibiting it. *Brucella* infection in the decidua disrupts this equilibrium, impairing migratory capacity. While low-dose neutralization of CXCL8 and CCL2 restores migration, high-dose blockade further reduces it, even under non-infected conditions. These results underscore the critical role of finely tuned chemokine signaling in trophoblast migration and suggest that infection-induced imbalances may contribute to placental dysfunction.

### 2.7. Conditioned Medium from Infected Decidualized Stromal Cells Impairs Trophoblast Invasion

Trophoblast invasion into the decidua is a critical process for proper placental development and pregnancy progression, as it ensures adequate maternal–fetal exchange and vascular remodeling. Disruptions in this process are associated with pregnancy complications. Given that the secretome of *Brucella*-infected decidual stromal cells induces a proinflammatory response in trophoblasts and impairs their migration, we next evaluated whether it also affects their invasive capacity.

Swan-71 cells were incubated for 1 h with CM from infected or non-infected decidual cells. Following incubation, the cells were seeded onto a protein matrix-coated membrane placed in the upper compartment of a Transwell system. The cultures were maintained for 18 h with continuous exposure to CM in the upper compartment, while the lower compartment was supplemented with DMEM/F12 containing 10% FBS to promote invasion. After stimulation, cells retained on the membrane were stained and quantified using microscopy-based image analysis.

**Figure 7:**
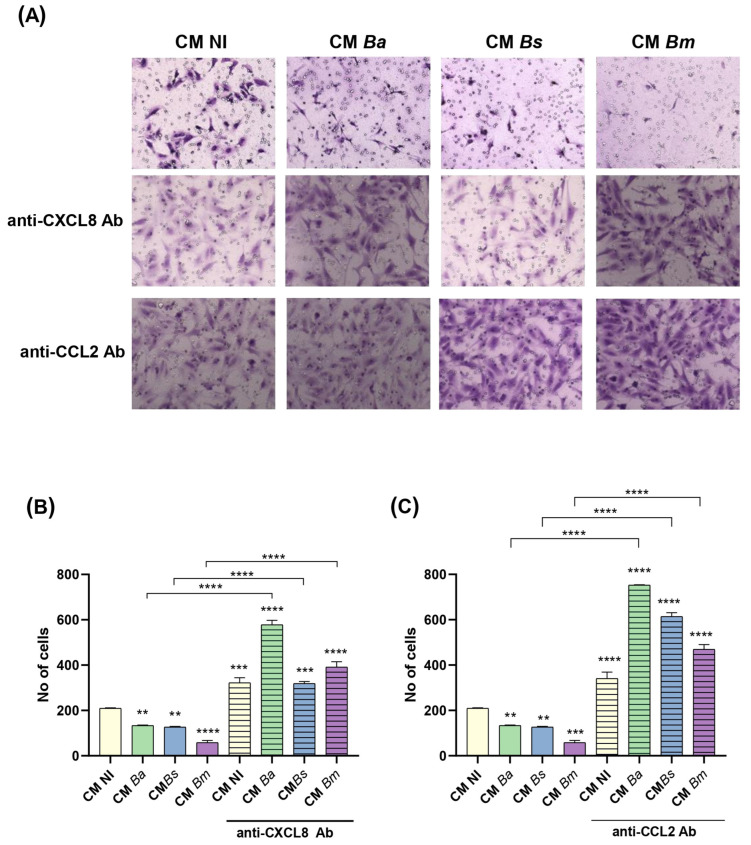
Conditioned medium from infected decidualized stromal cells impairs trophoblast invasion capacity. Swan-71 trophoblasts were incubated for 1 h with conditioned medium (CM) from decidual cells infected with *B. abortus* (*Ba*), *B. suis* (*Bs*), or *B. melitensis* (*Bm*), or from non-infected cells (NI). Then, trophoblasts were seeded onto a protein matrix-coated membrane placed in the upper compartment of a Transwell system and were maintained with continuous exposure to CM. The lower compartment was supplemented with DMEM/F12 containing 10% FBS to promote invasion. At 18 h of culture, the cells retained on the membrane were stained and quantified using microscopy-based image analysis. The same experiment was performed with pre-treatment of CM from both infected and non-infected cells with neutralizing antibodies against CXCL8 (**A**,**B**) or CCL2 (**A**,**C**). Representative 40× images of Swan-71 cell invasion assays treated with CM *Ba*, *Bs*, *Bm*, or CM NI, with or without neutralizing antibodies (**A**). Quantification of migrating cells (Nº of cells on the membrane) is shown as the mean ± SD of three independent experiments performed in duplicate (**B**,**C**). Asterisks over bars indicate differences versus the CM NI condition whereas asterisks over lines indicate differences between antibody-treated and untreated conditions (** *p* < 0.01; *** *p* < 0.001; **** *p* < 0.0001).

As shown in Figure 7A,B, stimulation with CM from *Brucella* spp.-infected decidual stromal cells (MOI 250) significantly reduced the number of invading trophoblasts compared to CM NI. Among the three *Brucella* species tested, CM from *B. melitensis*-infected decidual cells had the strongest inhibitory effect on trophoblast invasion. Moreover, pre-treatment of CM from both infected and non-infected cells with neutralizing antibodies against CXCL8 or CCL2 significantly increased trophoblast invasion compared to their respective untreated CM (Figure 7A,B). This suggests that physiological levels of CXCL8 and CCL2 are required to support proper trophoblast invasion, whereas an excessive production of these chemokines induced by *Brucella* infection of decidua cells disrupts this process. These findings indicate that *Brucella* spp. infection alters the secretome of decidual stromal cells, leading to an overproduction of CXCL8 and CCL2, which in turn impairs trophoblast invasive capacity.

### 2.8. Brucella Infection of Decidualized Stromal Cells Impairs Their Chemotactic Capacity for Trophoblasts

The above results demonstrated that exposure to CM from *Brucella*-infected decidual cells induces a proinflammatory response in trophoblasts, impairing both their migration and invasive capacity. Given the essential role of chemotactic signals in directing trophoblast movement, we next evaluated whether *Brucella* infection affects the chemotactic capacity of decidual stromal cells, potentially contributing to defective trophoblast invasion.

**Figure 8:**
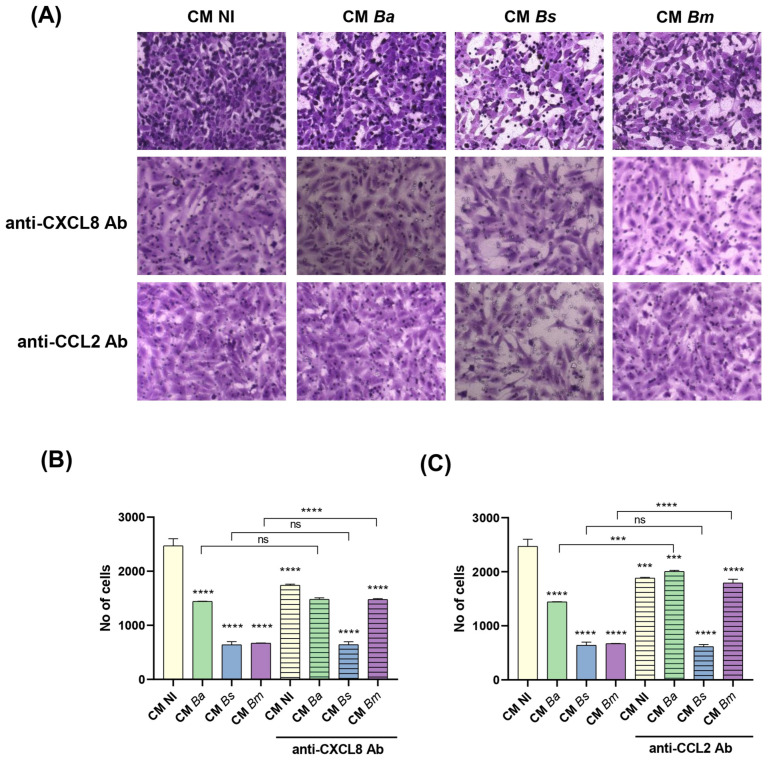
*Brucella* infection of decidualized stromal cells impairs their chemotactic capacity for trophoblasts. Swan-71 cells suspended in medium with 2% FBS were seeded onto a protein matrix-coated membrane placed in the upper compartment of a Transwell system. Conditioned media (CM) from decidual stromal cells infected with *B. abortus* (*Ba*), *B. suis* (*Bs*), or *B. melitensis* (*Bm*) or from non-infected cells (NI) were placed in the lower compartment to evaluate chemotactic activity. After 6 h of incubation, cells retained on the membrane were stained and quantified using microscopy-based image analysis. The same experiment was performed with pre-treatment of CM from both infected and non-infected cells with neutralizing antibodies against CXCL8 (**B**) or CCL2 (**C**). Representative images showing a 40× magnification of Swan-71 cells migration treated with CM *Ba*, *Bs*, *Bm*, or CM NI with and without neutralizing antibodies (**A**). The number of cells in the membrane (migrating cells) are expressed as mean ± SD of three independent experiments performed in duplicate (**B**,**C**). Asterisks over groups indicate differences versus the CM NI condition, while asterisks over lines indicate differences between antibody-treated and untreated conditions (*** *p* < 0.001; **** *p* < 0.0001; ns: non-significant).

Swan-71 cells were suspended in DMEM/F12 with 2% FBS and were seeded onto a protein matrix-coated membrane placed in the upper compartment of a Transwell system. CM from infected or non-infected decidual stromal cells was placed in the lower compartment to evaluate chemotactic activity. After 6 h of incubation, cells retained on the membrane were stained and quantified using microscopy-based image analysis. As shown in Figure 8A,B, stimulation with CM from *Brucella*-infected decidual stromal cells (MOI 250) significantly reduced trophoblasts migration compared to CM NI. Among the three *Brucella* species tested, CM *Bm* and CM *Bs* exhibited the strongest inhibitory effect on trophoblast chemotaxis (Figure 8).

To further assess the role of specific chemokines in this process, CM were pre-treated with neutralizing antibodies against CXCL8 or CCL2. CXCL8 neutralization in CM *Bm* significantly increased trophoblast migration compared to the respective untreated CM. However, blocking CXCL8 in CM *Ba* or CM *Bs* did not significantly alter trophoblast migration (Figure 8A). Interestingly, CXCL8 neutralization in CM NI led to a reduction in trophoblast migration compared to its untreated control, suggesting that physiological levels of CXCL8 are necessary for optimal chemotaxis.

Similarly, CCL2 neutralization in CM *Ba* and CM *Bm* resulted in increased trophoblast migration but had no significant effect on migration when applied to CM *Bs* (Figure 8B). Notably, as observed with CXCL8 blockade, CCL2 neutralization also reduced trophoblast migration induced by CM NI compared to the untreated control (Figure 8B), further supporting the idea that physiological levels of these chemokines are required for proper trophoblast chemotaxis. However, an excess of either CXCL8 or CCL2, as may be induced by *Brucella* infection, may result in impaired trophoblast migration. In addition, these results suggest that, particularly in the case of CM *Bs*, whose inhibitory effect on trophoblast chemotaxis was not reversed by CXCL8 or CCL2 neutralization, other infection-induced mediators not evaluated in this study may be involved in the modulation of this chemotactic process.

### 2.9. Conditioned Medium from Infected Decidualized Stromal Cells Impairs Trophoblast Tubulogenesis

After initial implantation, extravillous cytotrophoblasts invade the uterine wall and maternal spiral arteries, undergoing a specialized epithelial-to-endothelial transition known as pseudo-vasculogenesis [42], which is a form of tubulogenesis. This process is essential for placental development and successful pregnancy progression. As the secretome of *Brucella*-infected decidualized stromal cells was found to modify several trophoblasts functions, including migration and invasion capacity, we hypothesized that it may also disrupt trophoblast tubulogenesis by dysregulating key signaling molecules. To assess this, we evaluated the ability of trophoblasts to form tubular structures in response to stimulation with CM from infected and non-infected decidual cells.

Swan-71 cells were preincubated with CM *Ba*, CM *Bm*, CM *Bs*, or CM NI for 1 h and were then seeded on a protein matrix (Geltrex) in Transwell filters (8 µm pore size). Cells were then incubated for a further 6 h in the presence of the respective CM. After incubation, images were captured using a microscope to assess the number of master segments, master junctions, and meshes, which are key parameters of tubulogenesis.

**Figure 9:**
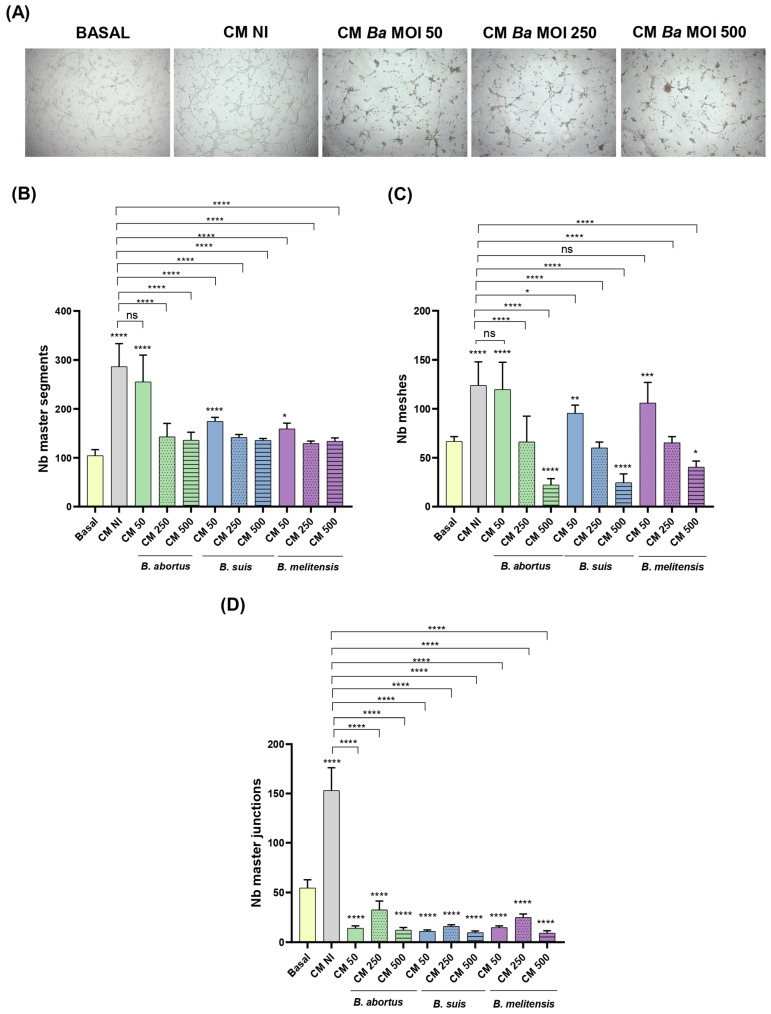
Factors produced by *Brucella*-infected decidualized stromal cells impair trophoblast tubulogenesis. Swan-71 cells were seeded on a protein matrix (Geltrex) and incubated for 6 h with conditioned media (CM) from *B. abortus*, *B. suis*, or *B. melitensis*-infected decidual cells (MOI 50, 250 or 500) or from non-infected cells (NI), or were left untreated (Basal). After incubation, images were captured using a microscope to assess the number of master segments (**B**), meshes (**C**), and master junctions (**D**). Representative images showing a 40× magnification of tubulogenesis of Swan-71 cells treated with CM *Ba* at different MOIs or CM NI (**A**). The results are expressed as mean ± SD of three independent experiments performed in duplicate. Asterisks over the bars indicate differences versus the Basal condition whereas asterisks over lines indicate differences versus CM NI (* *p* < 0.05, ** *p* < 0.01; *** *p* < 0.001; **** *p* < 0.0001; ns: non-significant). Representative images of the assay are shown in panel (**D**).

As expected, stimulation of trophoblasts with CM NI increased the number of master segments and meshes compared to the unstimulated control (Basal) (Figure 9A,B). Similarly, stimulation with CM from decidual cells infected at the lowest MOI (MOI 50) of the tested *Brucella* species significantly increased the number of master segments and meshes compared to the basal control, although to a significantly lower extent than CM NI. In contrast, CM from decidual cells infected at the higher MOIs (MOI 250 and 500) did not induce this increase, suggesting an MOI-dependent inhibitory effect of *Brucella* on tubulogenesis (Figure 9A,B). Stimulation with CM NI also increased the number of master junctions compared to the unstimulated control (Figure 9C). In contrast, CM from cells infected with *Brucella* species at all tested MOIs did not increase the number of master junctions compared to the basal control. Representative images are shown in Figure 9D. These findings suggest that CM from *Brucella*-infected decidualized stromal cells impairs trophoblast tubulogenesis in an MOI-dependent manner. While CMs from low MOI-infected decidual cells still promote some tubulogenesis, higher MIs do not enhance this process and may even inhibit it. These results highlight the potential impact of *Brucella*-induced dysregulation of stromal signaling on placental development.

**Figure 10:**
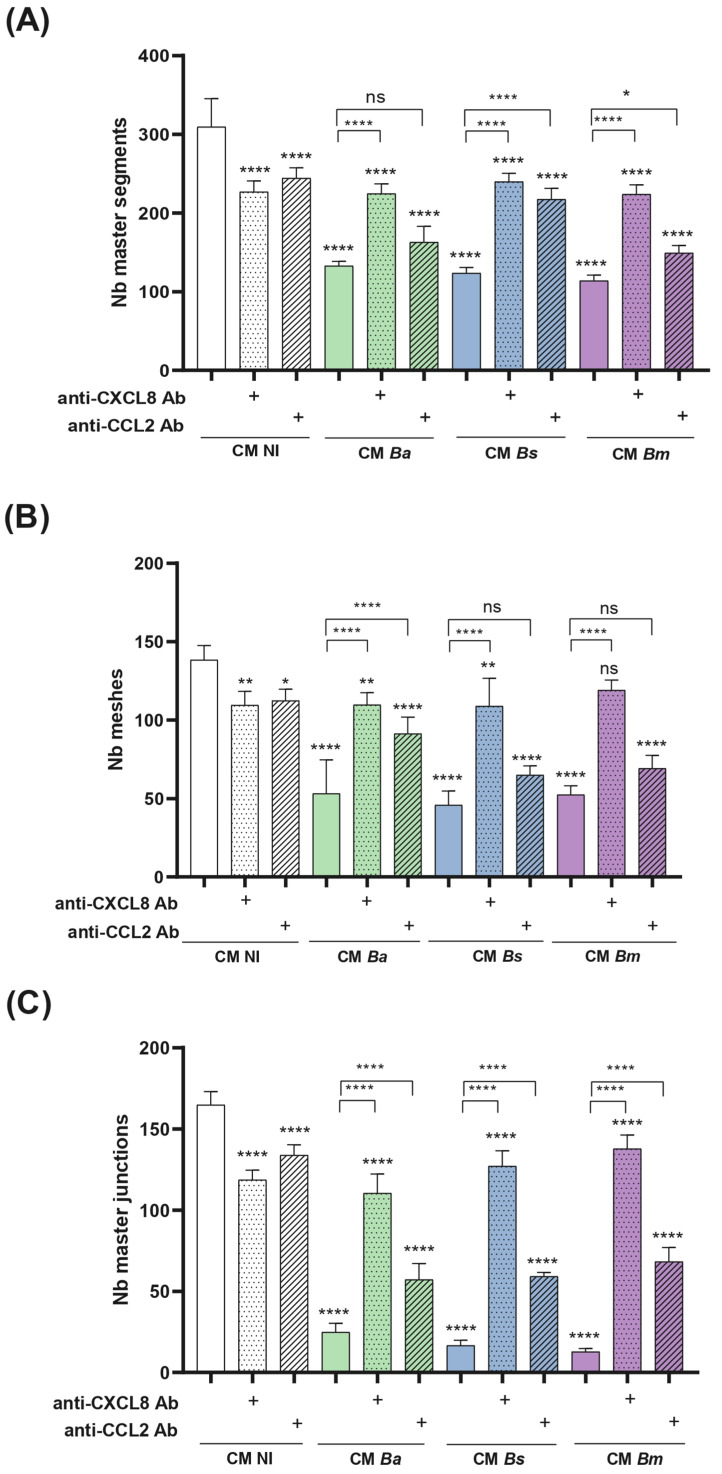
Proinflammatory chemokines induced by *Brucella* infection in decidual cells contribute to the reduced ability of trophoblasts to form tubular structures. A tubulogenesis assay was performed as indicated in Figure 9, but conditioned media (CM) from *Brucella*-infected decidual cells were preincubated with neutralizing antibodies (Ab) against CXCL8 or CCL2 before addition to Swan-71 trophoblasts. After incubation, images were captured using a microscope to assess the number of master segments (**A**), meshes (**B**), and master junctions (**C**). The results are expressed as mean ± SD of three independent experiments performed in duplicate. Asterisks over bars indicate differences versus the untreated CM NI (white bar), whereas asterisks over lines indicate differences between each Ab-treated and the untreated CM for each *Brucella* species (* *p* < 0.05; ** *p* < 0.01; **** *p* < 0.0001; ns: non-significant).

To assess whether proinflammatory chemokines induced by decidual cell infection contribute to the reduced ability of trophoblasts to form tubular structures, we repeated the tubulogenesis assays by pre-incubating the CM with neutralizing antibodies against CXCL8 and CCL2. As shown in Figure 10, treatment with anti-CXCL8 or anti-CCL2 antibodies increased the number of master segments, meshes, and junctions in cells stimulated with CM from *Brucella*-infected cells (MOI 250) compared to the respective untreated CM. Notably, neutralizing these chemokines in CM NI also significantly reduced all tubulogenesis parameters compared to the untreated CM NI, highlighting the dual role of inflammatory signaling in trophoblast tubulogenesis.

## 3. Discussion

Trophoblast invasion of the maternal endometrial stroma and the remodeling of spiral arteries mediated by these cells are fundamental processes in early placental development. Disruptions in any of these steps can compromise pregnancy progression and lead to severe gestational pathologies. Decidual stromal cells play a pivotal role in modulating trophoblast function during implantation. These maternal cells create a finely tuned microenvironment that supports trophoblast migration, invasion, and differentiation by secreting cytokines, chemokines, growth factors, and extracellular matrix components. For example, factors secreted by decidualized endometrial stromal cells enhance trophoblast invasion by upregulating matrix metalloproteinases (MMPs) and integrins [43]. Moreover, decidual stromal cells actively shape the local immune landscape, contributing to immune tolerance and tissue remodeling, both essential for successful placentation.

Using the first-trimester placental trophoblast cell line Swan-71 and the telomerase-immortalized human endometrial stromal cell line T-HESC as experimental models, we demonstrated that infection with virulent *Brucella* species impairs endometrial decidualization and induces an exacerbated inflammatory microenvironment, ultimately disrupting trophoblast functionality.

We had previously reported that *B. abortus* infection of already decidualized stromal cells did not alter their decidual phenotype [32]. However, the present study shows that prior *Brucella* infection of stromal endometrial cells attenuates the subsequent decidualization process, reflected in reduced PRL levels, in a MOI-dependent manner across all virulent *Brucella* species tested.

This observation is particularly relevant because animal models show that the uterus can serve as a reservoir for *Brucella* persistence and replication even outside pregnancy, and chronic *Brucella* infection has been linked to infertility [34]. Thus, previously infected stromal cells may undergo incomplete or defective decidualization, impairing cross talk with trophoblasts and contributing to gestational complications associated with brucellosis. Interestingly, Lei et al. [33] recently demonstrated that purified LPS from *B. suis* S2 also disrupts decidualization, leading to implantation failure.

Importantly, we found that the secretome of infected and later decidualized T-HESCs showed increased levels of CXCL8 and CCL2, but not IL-6, as compared to non-infected cells undergoing the same decidualization treatment. This increased production of CXCL8 and CCL2 is in line with our previous findings in *B. abortus*-infected non-decidualized T-HESC or in T-HESC cells infected after decidualization [32], and also agrees with previous studies that showed that both decidual and endometrial stromal cells express TLRs and NLRs and respond to microbial molecules with the production of inflammatory cytokines [26,27,44]. Here we also found that CM from these infected and later decidualized T-HESC cells stimulated Swan-71 trophoblasts to secrete IL-6, CXCL8, and CCL2 as compared to CM from non-infected cells. The influence of factors produced by decidual stromal cells (and other decidual cells) on diverse trophoblast functions, including the secretion of cytokines and chemokines, has been extensively reported [38,45,46]. This study shows that preinfection of decidual stromal cells with *Brucella* modifies the secretome of trophoblasts, promoting a proinflammatory profile, although the factors involved were not identified. Notably, these infection-driven changes in the decidual microenvironment correlated with reduced acquisition of invasive phenotypes by trophoblasts, suggesting that an altered cytokine milieu negatively affects trophoblast behavior.

A key step in implantation is thus the coordinated migration and invasion of trophoblast cells through the maternal tissue. Among the various maternal-derived factors modulating this process, CXCL8 and CCL2 play a critical role. Decidual stromal cells and uterine natural killer cells are prominent sources of CXCL8 within the maternal–fetal interface, and its receptors are highly expressed on extravillous trophoblasts (EVTs) [47]. Proper CXCL8 signaling is considered essential for a successful pregnancy, as diminished levels of CXCL8 or its receptors have been linked to recurrent pregnancy loss [48]. Our group [49] and others [50,51] have shown that exogenous CXCL8 stimulates MMP secretion, thereby promoting the migration and invasion of HTR-8 trophoblast cells. However, under pathological conditions, excessive CXCL8 levels may exert detrimental effects, shifting from a stimulatory to an inhibitory role in trophoblast behavior [49,50]. Consistent with these previous observations, we demonstrate here that neutralization of CXCL8 partially reverses the ability of CM from *Brucella*-infected and decidualized T-HESCs to inhibit trophoblast migration and invasion. Interestingly, the highest concentration of neutralizing anti-CXCL8 antibody also reduced migration induced by CM from non-infected decidualized T-HESCs, reinforcing the idea that a fine-tuned balance of CXCL8 is critical for optimal trophoblast function.

Moreover, our study shows that trophoblast migration was also restored when CCL2 in the CM from infected and decidualized T-HESCs was neutralized. CCL2 is secreted by decidual tissue during early pregnancy, where its production is induced by cytokines, estrogens, progesterone, and hCG, and is regulated in an autocrine manner via the ERK/MAPK pathway through its receptor CCR2 [52,53,54]. Variations in CCL2 levels can influence both normal pregnancy progression and pathological outcomes [54,55]. Although CCR2 mRNA has been detected in first-trimester bovine trophoblasts [56], the expression of CCR2 in human trophoblasts remains uncertain. Nevertheless, CCL2 can promote cell migration through the CCR4 receptor [57]. CCR4 expression has been specifically detected in invasive EVTs where it mediates migration [58]. Thus, the CCL2/CCR4 axis could partially mediate the migratory response seen upon treatment with CM from *Brucella*-infected decidual cells.

Beyond direct effects, CCL2 exerts indirect trophoblast regulation by recruiting macrophages expressing G-CSF, which promote EVT growth and invasion [59], guiding Th17 cells that produce IL-17 [56], and suppressing COX-2-associated oxidative stress, creating a favorable implantation environment. Additionally, since Swan-71 cells stimulated with CM from infected decidual cells also secreted CCL2 and CXCL8, we cannot exclude autocrine effects of these chemokines on trophoblast migration.

Although IL-6 has been shown to enhance trophoblast migration and invasion [60], we observed no significant increase in IL-6 levels following decidual infection. However, excessive IL-6 produced by Swan-71 cells in response to CM from infected decidual cells could still contribute to the inhibition of migratory and invasive capacity.

Our data further show that previous *Brucella* infection of stromal endometrial cells not only modified the cytokine profile but also affected the chemotactic response of trophoblast cells, reducing their directed migration toward the CM. These findings highlight that beyond impairing intrinsic trophoblast invasive capacity, infection-driven alterations in the decidual secretome compromise its chemotactic and supportive functions towards trophoblasts, further disrupting the finely orchestrated maternal–trophoblast interactions essential for successful implantation and placentation. Overall, these findings indicate that *Brucella*-induced alterations in the levels of factors secreted by decidual cells, including CXCL8 and CCL2, impair the migration and invasion capacities of trophoblasts. Literature supports the requirement of fine-tuned levels of decidual factors to promote these trophoblastic functions, which could be altered in the context of infections. However, we have been unable to find reports of impaired trophoblastic functions by decidual cells, similar to those seen here for *Brucella* spp., in the context of infections by other pathogens. In this sense, this study seems to be the first to show this possibility.

Following the initial stages of implantation, extravillous cytotrophoblasts progressively invade the uterine wall and maternal spiral arteries, undergoing a specialized epithelial-to-endothelial transition known as vascular mimicry or pseudo-vasculogenesis, which is essential for proper placental vascular remodeling [42]. The remodeling process can be seen as a form of tubulogenesis, where new tubular structures (the remodeled spiral arteries) are formed. Our results demonstrate that CM from *Brucella*-infected decidualized T-HESC significantly impairs the tubulogenic capacity of Swan-71 trophoblast cells, whereas CM from non-infected THESC promotes tubule formation. This inhibitory effect was MOI-dependent across all *Brucella* species tested. Therefore, *Brucella* infection may also hamper a proper vascular remodeling of the placenta, leading to pregnancy complications.

Although numerous studies have shown that CCL2 promotes angiogenesis in endothelial cells [61], a recent report demonstrated that thrombin can inhibit tube formation by HTR-8 trophoblast cells through the induction of CCL2 production [62]. Consistent with these findings, our study shows that CCL2 present in CM from infected decidualized T-HESC negatively and partially regulates the tube-forming capacity of Swan-71 cells. While CCL2 is essential for maintaining a successful pregnancy, elevated CCL2 levels have been associated with pathological conditions such as preeclampsia [54]. Therefore, we speculate that the negative impact of CM from the infected decidua on Swan-71 tubulogenesis and spiral artery remodeling may critically depend on the local concentration of CCL2.

Another key pro-angiogenic factor involved in trophoblast function is CXCL8. Our results reveal that CXCL8 present in the CM of infected decidualized T-HESC plays a central role in regulating tubulogenesis, as neutralization of CXCL8 in this CM partially restores tubule formation to levels comparable to those observed with CM from non-infected controls. These findings suggest that, although physiological levels of CXCL8 promote trophoblast migration, invasion, and tubulogenesis, consistent with its well-established pro-angiogenic effects in both endothelial and trophoblast cells [63,64], excessive or deregulated CXCL8 under pathological conditions may disrupt these processes, contributing to defective spiral artery remodeling and compromised placental development. Interestingly, a study in rats with intrauterine infection by *Porphyromonas gingivalis* suggested that disturbances in the decidual stroma, including altered cytokine levels, were involved in the impairment of spiral artery remodeling [65].

Overall, our study highlights the dual and context-dependent roles of CXCL8 and CCL2 signaling in trophoblast biology, underscoring the importance of maintaining a finely tuned balance of pro-angiogenic factors at the maternal–fetal interface to ensure successful placentation.

Previous studies have established that the decidua plays a pivotal role in supporting implantation by facilitating trophoblast outgrowth. Decidualized human endometrial stromal cells are notably more effective in this role compared to their non-decidualized counterparts, largely due to their capacity to secrete matrix metalloproteinases (MMPs), migrate, and encase the blastocyst, thereby regulating the extent of its invasion [41,66]. To investigate how *Brucella* infection affects endometrial receptivity, we developed an in vitro implantation model by laying blastocyst-like spheroids derived from Swan-71 trophoblasts on either *Brucella*-infected or uninfected decidualized stromal cells. Our results revealed that *Brucella* infection significantly impaired trophoblast spreading over the stromal monolayer, with *B. melitensis* exerting the most pronounced inhibitory effect. Of note, previous studies have shown that activation of TLRs in the endometrium reduces the adhesion of trophoblasts spheroids, a fact that has been linked to infection-driven implantation failures [67,68]. These results suggest that *Brucella* infection of the decidua may also lead to implantation failures.

Progesterone has several key roles in pregnancy maintenance, including modulation of maternal immune responses, reduction of uterine contractility, improvement of utero-placental circulation, promotion of extravillous trophoblasts invasion to the decidua, and stimulation of proliferation and differentiation of endometrial stromal cells [19,37]. After placental development, progesterone is produced by trophoblasts under the control of several factors including estrogen, hCG, and corticotropin-releasing hormone (CRH). GlycodelinA, a glycoprotein secreted by decidual cells, has been shown to stimulate progesterone and hCG production in different trophoblast populations [40,69]. Here we found that CM from decidualized T-HESC cells stimulates progesterone production in trophoblasts, but this stimulatory effect is significantly reduced when T-HESC are infected with *Brucella* prior to decidualization. While the mechanisms involved in this reduced progesterone production remain to be established, these results suggest that *Brucella* infection may negatively impact the ability of decidual cells to stimulate progesterone secretion by trophoblasts, thus reducing the gestation-promoting effects of this hormone.

## 4. Materials and Methods

### 4.1. Brucella spp. Growth Conditions

*B. abortus* 2308, *B. suis* 1330 and *B. melitensis* 16M (wild type strains, obtained from our collection) were grown in tryptic soy broth at 37 °C with agitation. Bacterial cells were washed twice with sterile phosphate buffer saline (PBS), and the respective inocula were adjusted to the desired concentration based on optical density (OD) readings. Aliquots of each inoculum were plated on tryptic soy agar (TSA) and incubated at 37 °C to determine the actual number of colony-forming units (CFU) per mL. All live *Brucella* manipulations were performed in biosafety level 3 facilities.

### 4.2. Cell Lines

The human endometrial stromal cell line used in this study (T-HESC, kindly provided by Dr. Andrea Randi, University of Buenos Aires) was derived from normal stromal cells through immortalization by telomerase (hTERT) transfection, and conserved the characteristics of the regular endometrial cells [70]. T-HESC were maintained in DMEM-F12 (without phenol red) supplemented with 10% fetal bovine serum, 100 U/mL penicillin, 50 µg/mL streptomycin, 2 mM glutamine, 50 mM sodium pyruvate, and 500 ng/mL puromycin. For infection assays, cells were cultured for 24 h in antibiotic-free culture medium.

The human trophoblastic cell line Swan-71, obtained from normal first trimester trophoblasts through immortalization by hTERT transfection, was kindly provided by Dr. Gil Mor (Wayne State University). These cells conserve most phenotypical characteristics of primary trophoblasts as well as their biological responses [71]. Swan-71 cells were cultured in DMEM/F-12 supplemented with 10% FBS, 100 U/mL penicillin, 50 µg/mL streptomycin, 2 mM glutamine, and 50 mM sodium pyruvate.

### 4.3. Cellular Infections

T-HESC cells were dispensed at 5 × 10^4^ cells/well in 24-well plates, and were cultured for 24 h in antibiotic-free medium at 37 °C in a 5% CO_2_ atmosphere. Cells were infected with *B. abortus*, *B. melitensis*, or *B. suis* at a MOI of 50, 250, or 500 for 2 h. After dispensing the bacterial suspension, the plates were centrifuged (10 min at 400× *g*) and then incubated for 2 h at 37 °C in a 5% CO_2_ atmosphere. Non-internalized bacteria were eliminated by several washes with medium alone followed by incubation in medium supplemented with 100 µg/mL gentamicin and 50 µg/mL streptomycin. After that, cells were washed and then incubated with culture medium without antibiotics.

### 4.4. Decidualization of T-HESC Endometrial Cells

T-HESC were infected or not with *B. abortus*, *B. suis*, or *B. melitensis* at different MOIs (50, 250, 500) as described above, and 24 h later cells were subjected to decidualization treatment as described previously [72], with minor modifications. Briefly, T-HESC (5 × 10^4^ cells/well) were cultured in DMEM/F12 2% FBS with medroxyprogesterone acetate (MPA, 1 µM) and dibutyryl cAMP (0.5 mM). The culture medium was replaced every 48 h to maintain hormonal stimulation. Cells were maintained at 37 °C in a humidified atmosphere with 5% CO_2_ throughout the experiment. Decidualization status was evaluated at 1, 2, 4, and 6 days after the initiation of treatment by measuring prolactin levels in the culture supernatants using a sandwich ELISA kit (R&D Systems, Minneapolis, MN, USA), following the manufacturer’s instructions.

### 4.5. Citotoxicity

To assess cell integrity, LDH release from infected and control decidualized T-HESC cultures was quantified at 6 days p.i. using the CytoTox 96 assay (Promega, Madison, WI, USA) following the manufacturer’s instructions, and was expressed as a ratio to the maximum LDH obtained by hypotonic lysis.

### 4.6. Preparation of CM from Infected and Decidualized T-HESC

CM were obtained from T-HESC that had undergone sequentially the infection and decidualization procedures described above. After 8 days of decidualization treatment, culture supernatants were collected and subsequently filtered through 0.22 µm pore-size filters to ensure sterility. To obtain control CM, mock-infected T-HESC were subjected to the same decidualization protocol to simulate the infection and decidualization conditions without bacterial exposure. The levels of CXCL8 and CCL2 were measured in these CM using commercial ELISA kits (R&D Systems).

### 4.7. Cytokine Response of Trophoblasts Stimulated with T-HESC CM

To evaluate the impact of a *Brucella*-infected decidua on the cytokine response of adjacent trophoblasts, Swan-71 cells (5 × 10^4^ cells/well) were stimulated with CM from infected and NI decidualized stromal cells. The CM was applied at 1:2 and 1:5 dilution. After 24 and 48 h of stimulation, culture supernatants were collected to measure L-6, CXCL8, and CCL2 levels using commercial ELISA kits (R&D Systems). To determine the cytokine production specifically attributable to the stimulated trophoblasts, baseline cytokine levels present in the corresponding CM were subtracted from the measured levels in the stimulated cultures.

### 4.8. Progesterone Response of Trophoblasts Stimulated with T-HESC CM

Swan-71 trophoblasts (5 × 10^4^ cells/well) were stimulated for 48 h with CM *Ba*, *Bs*, or *BM* or from non-infected decidualized cells. Culture supernatants of the stimulated trophoblasts were harvested to determine progesterone levels using a commercial ELISA kit (Cayman Chemical Company, Ann Arbor, MI, USA) following the instructions of the manufacturer.

### 4.9. Functional Response of Trophoblasts Stimulated with T-HESC CM

(a) Migration. The effect of CM from infected and non-infected decidualized stromal cells on the migration ability of trophoblasts was evaluated using the scratch test essentially as described by Rattila et al. [73]. Swan-71 cells were plated at 5 × 10^4^ cells/well in culture medium without antibiotics to allow the formation of a confluent layer. The following day, the culture medium was carefully removed and cells were gently washed with DMEM-F12. A vertical scratch was performed through the monolayer using a pipette tip, and cells were cultured in the presence of CM from infected and non-infected decidualized stromal cells. Swan-71 cells cultured in DMEM/F-12 supplemented with 10% FBS were used as a positive control of migration. Pictures were taken at time 0 and later on the same microscopic field at 18 h post-stimulation. Each wound area in duplicate wells was measured with ImageJ software. The degree of wound closure was calculated as: [(area time 0 h − area time = 18 h)/area time = 0 h] × 100. The independent experiments were repeated three times.
(b) Invasion. Trophoblast invasive capacity was evaluated as described by Rattila et al. [74]. A Geltrex matrix (0.6 mg/mL in DMEM-F12) was applied to Transwell inserts (8 µm pore size) in 24-well plates to simulate the basal membrane. Swan-71 cells (3 × 10^4^) were pre-treated for 1 h with CM (1:2 dilution) from non-infected or *Brucella*-infected decidualized stromal cells (MOI 250), then seeded in the upper chamber on top of the Geltrex matrix. CM was maintained throughout the assay in the upper chamber.

In the lower chamber of the insert, 500 µL of DMEM-F12 with 10% FCS were dispensed. After 18 h of culture the medium was removed and the matrix was cleaned with a cotton swab. Transwells were very carefully washed with PBS and were fixed with PFA 4% for 15 min. After a further wash, the Transwells were stained with crystal violet for 15 min. The excess of colorant was removed with distilled water and the membrane was carefully cleaned with a swab on the upper side, without touching the lower chamber to avoid the removal of cells that crossed the matrix and the membrane. The membranes of inserts were analyzed using an EVOS microscope (Thermo Fisher, Waltham, MA, USA) to determine the number of cells that crossed the membranes. The positive control wells included 10% FBS as the attractive stimulus to check the functionality of cells.

(c) Tubulogenesis. This assay was performed essentially as described by Rattila et al. [73]. A Geltrex matrix (9 mg/mL in DMEM-F12) was dispensed in a 96-well plate. When gelification was complete, Swan-71 cells (2 × 10^4^/well) previously stimulated for 1 h with CM *Ba*, *Bs*, or *Bm* MOI 50, 250, or 500) or NI stromal decidualized cells were dispensed. After 6 h the wells were imaged using an EVOS microscope (Thermo Fisher) to evaluate the number of master junctions, master segments, and meshes using ImageJ software and the Angiogenesis Analyser plugin. CM were kept during the whole assay.

### 4.10. Evaluation of the Chemotactic Effect of CM from Decidualized Stromal Cells on Trophoblasts

To assess the chemotactic effect of CM from decidualized stromal cells, either infected with *Brucella* or not, a Transwell-based migration assay was performed. This assay aims to determine whether soluble factors present in the CM can promote the directed migration of trophoblast cells. A similar protocol to the invasion assay described above was used, with key modifications to specifically evaluate chemotaxis. CM from infected (MOI 250, three *Brucella* species) or non-infected decidualized stromal cells was placed in the lower chamber of the Transwell inserts, while Swan-71 cells were seeded in the upper chamber on a Geltrex matrix without prior CM treatment. The assay was conducted for 6 h, with 2% FBS present in both chambers to control for serum-driven migration. The membranes of inserts were analyzed using an EVOS microscope (Thermo Fisher) to determine the number of cells retained in the membranes.

### 4.11. Neutralization of CXCL8 and CCL2 in CM

To test the role of CXCL8 and CCL2 in the effects produced by CM from infected or non-infected decidualized stromal cells, in some experiments these CM were preincubated for 1 h with neutralizing antibodies against these chemokine (R&D Systems) in two different concentrations (0.5 µg/mL and 1 µg/mL). The neutralizing antibodies in CM were maintained for the duration required for each functional assay in which they were used. In some experiments, CM were preincubated with mixtures of both antibodies.

### 4.12. Spheroids Adhesion to the Infected Decidua

In order to form blastocyst-like spheroids [75], Swan-71 cells were stained with VybrantTM DiD (Thermo Fisher Scientific) and were dispensed in a low-binding Petri dish at 2 × 10^4^ cells per 20 μL drop of medium (DMEM/F-12 supplemented with 2% FCS). Cells were incubated at 37 °C with agitation for 48 h. At this time, cells were observed under the optical microscope to check whether the spheroids had formed correctly.

T-HESC cells were plated at 1 × 10^4^ cells/well in 96-well plates, were infected (or not) with the tested *Brucella* species (MOI 50, 250, 500) and were later decidualized as described above. Trophoblasts spheroids were carefully laid on the decidualized cells using a pipette with a blunt end tip to avoid damaging the spheroids. Images were taken at time 0 (T0) and 24 h later (T24) using an EVOS microscope (Thermo Fisher), and were analyzed using ImageJ software. Spheroids areas were measured at both time points and results were expressed as relative spheroid outgrowth calculated as area T24/area T0.

### 4.13. Statistical Analysis

Each experiment was performed in duplicate on three separate occasions. The data are presented as the mean ± standard deviation (SD). Statistical analyses were performed using one-way ANOVA, followed by either Tukey’s post hoc test or Dunnett’s post hoc test, with GraphPad Prism 8.0 software.

## 5. Conclusions

Our findings demonstrate that infection of the stromal cells with virulent *Brucella* species exerts profound negative effects on the cellular or molecular dialogue in the maternal-fetal interface, impairing key processes required for successful implantation and placental development. By disrupting endometrial stromal cell decidualization, altering the local cytokine and chemokine milieu, and inhibiting trophoblast migration, invasion, and tubulogenesis, *Brucella* infection compromises the coordinated cellular interactions necessary for proper trophoblast function and spiral artery remodeling, as shown in this in vitro model of maternal–fetal interaction.

Importantly, our data reveal that while maternal-derived factors such as CXCL8 and CCL2 play essential roles in promoting trophoblast differentiation and vascular remodeling under physiological conditions, their excessive or dysregulated production in the context of infection paradoxically inhibits these processes, potentially contributing to defective placentation.

Overall, this study highlights the decidua as a critical target of *Brucella*-induced pathology and positions infection-driven defects in maternal stromal cells as key contributors to gestational complications. By elucidating the molecular and cellular mechanisms underlying these interactions, our work provides new insights into how *Brucella* infections compromise fertility and pregnancy outcomes, while also opening avenues for the development of targeted interventions to restore decidual–trophoblast cross talk and improve reproductive health in infected individuals.

## Data Availability

The raw data supporting the conclusions of this article will be made available by the authors on request.

## Supplementary Materials

The following supporting information can be downloaded at https://www.mdpi.com/article/10.3390/ijms26178189/s1.
