# Supplementary material for: Brucella-Induced Impairment of Decidualization and Its Impact on Trophoblast Function and Inflammatory Profile
Source: Int J Mol Sci. 2025 Aug 23;26(17):8189. doi: 10.3390/ijms26178189 (PMC12427681; doi:10.3390/ijms26178189)
Supplement: Supplementary file 1 [file ijms-26-08189-s001.zip › captions supplementary figures.pdf]

Figure S1. ***Brucella*** **pre-infection does not impact on prolactin production by human endometrial stromal cells at early time points after decidualization stimuli.** Endometrial stromal cells from the T-HESC line were infected with *B. abortus (Ba)*, *B. melitensis (Bm)*, or *B. suis* (*Bs*) at different multiplicities of infection (MOI) or were left uninfected (NI) as controls. At 24 h post-infection, both infected or non-infected cells were subjected to the decidualization protocol. Culture supernatants were collected at the indicated time points, and prolactin (PRL) levels were quantified by ELISA. Results are expressed as mean ± SD of three independent experiments performed in duplicate. ***p<0.0001 versus NI.

Figure S2. Conditioned medium from ***Brucella suis*****-infected decidual cells impairs trophoblast migration.** Swan-71 trophoblasts were dispensed at 5 x 10^4 cells/well and were grown until confluence. A scratch was performed in the culture with a pipette tip, and then cells were stimulated with conditioned medium from *B. suis*–infected and later decidualized THESC cells (CM *Bs*) or from non-infected but decidualized cells (CM NI). Stimulation with the corresponding CM was maintained during the whole assay. Swan-71 cells cultured in DMEM/F-12 supplemented with 10% FBS were used as a positive control of migration (+C). To evaluate wound closure, pictures were taken at time 0 and at 18 h post-stimulation on the same microscopic field. Pictures were processed using ImageJ software. The percentage of wound healing was calculated as: [(area time 0 h - area time = 18h) / area time = 0 h] x 100. In parallel experiments, CM *Bs* and CM NI were preincubated (or not) for 1 hour with two concentrations (0.5 and 1 µg/ml) of neutralizing antibodies against CXCL8 (A and B) or CCL2 (C and D) or a mixture of both (E and F) before performing the wound healing assay. Results are expressed as mean ± SD of three independent experiments performed in duplicate. Asterisks over bars indicate differences versus the +C condition, whereas asterisks over lines indicate differences between antibody-treated and untreated conditions (*p<0.05; ** p<0.01; *** p<0.001; **** p<0.0001; ns: non-significant).

Figure S3. Conditioned medium from ***Brucella melitensis*****-infected decidual cells impairs trophoblast migration.** Swan-71 trophoblasts were dispensed at 5 x 10^4 cells/well and were grown until confluence. A scratch was performed in the culture with a pipette tip, and then cells were stimulated with conditioned medium from *B. melitensis*–infected and later decidualized THESC cells (CM *Bm*) or from non-infected but decidualized cells (CM NI). Stimulation with the corresponding CM was maintained during the whole assay. Swan-71 cells cultured in DMEM/F-12 supplemented with 10% FBS were used as a positive control of migration (+C). To evaluate wound closure, pictures were taken at time 0 and at 18 h post-stimulation on the same microscopic field. Pictures were processed using ImageJ software. The percentage of wound healing was calculated as: [(area time 0 h - area time = 18h) / area time = 0 h] x 100. In parallel experiments, CM *Bm* and CM NI were preincubated (or not) for 1 hour with two concentrations (0.5 and 1 µg/ml) of neutralizing antibodies against CXCL8 (A and B) or CCL2 (C and D) or a mixture of both (E and F) before performing the wound healing assay. Results are expressed as mean ± SD of three independent experiments performed in duplicate. Asterisks over bars indicate differences versus the +C condition, whereas asterisks over lines indicate

differences between antibody-treated and untreated conditions (*$p<0.05$; ** $p<0.01$; *** $p<0.001$; **** $p<0.0001$; ns: non-significant).
