# Supplementary figures and images for: Brucella-Induced Impairment of Decidualization and Its Impact on Trophoblast Function and Inflammatory Profile
Source: Int J Mol Sci. 2025 Aug 23;26(17):8189. doi: 10.3390/ijms26178189 (PMC12427681; doi:10.3390/ijms26178189)

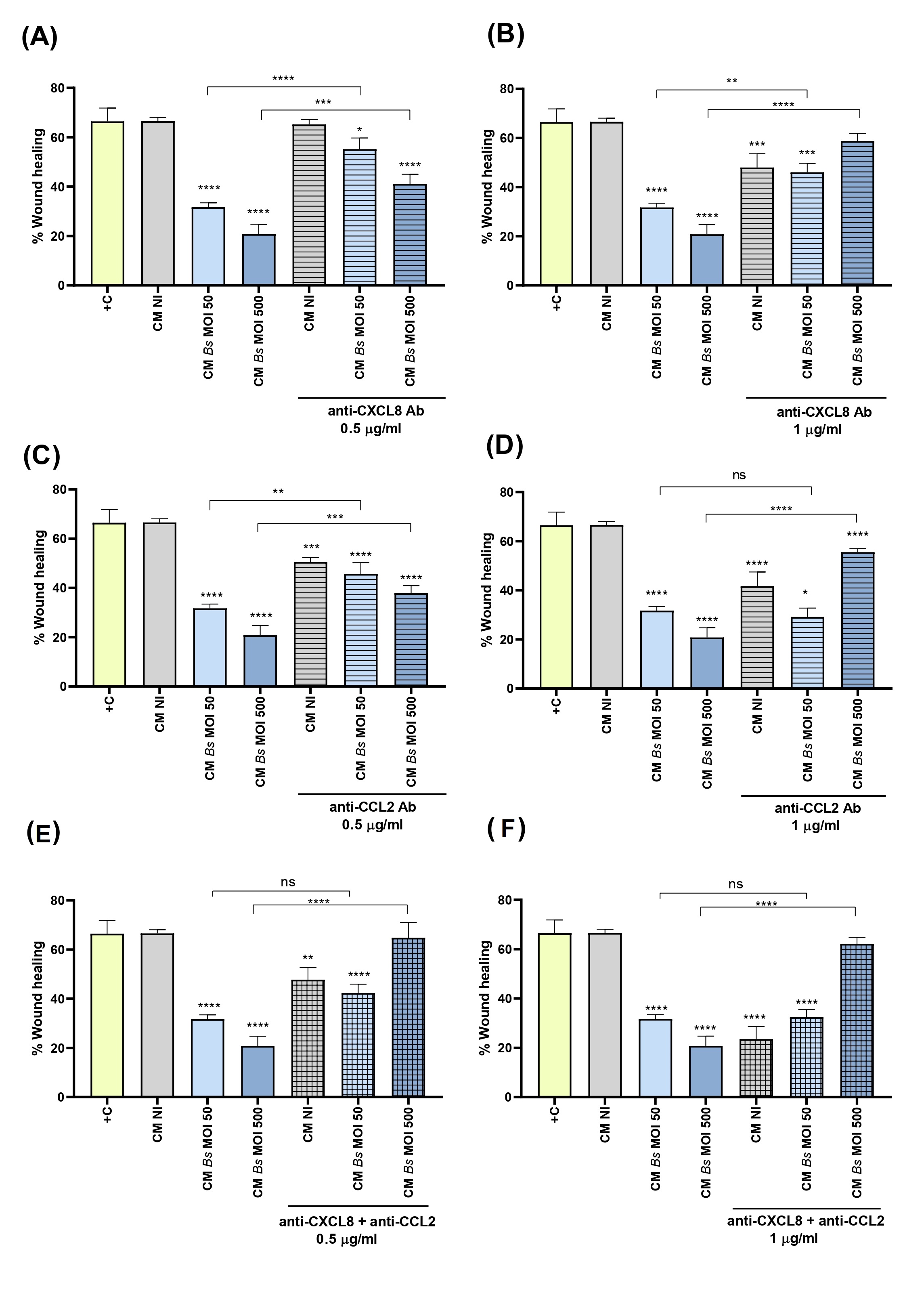

Supplement: Supplementary file 1 [file ijms-26-08189-s001.zip › Figure S2.jpg]

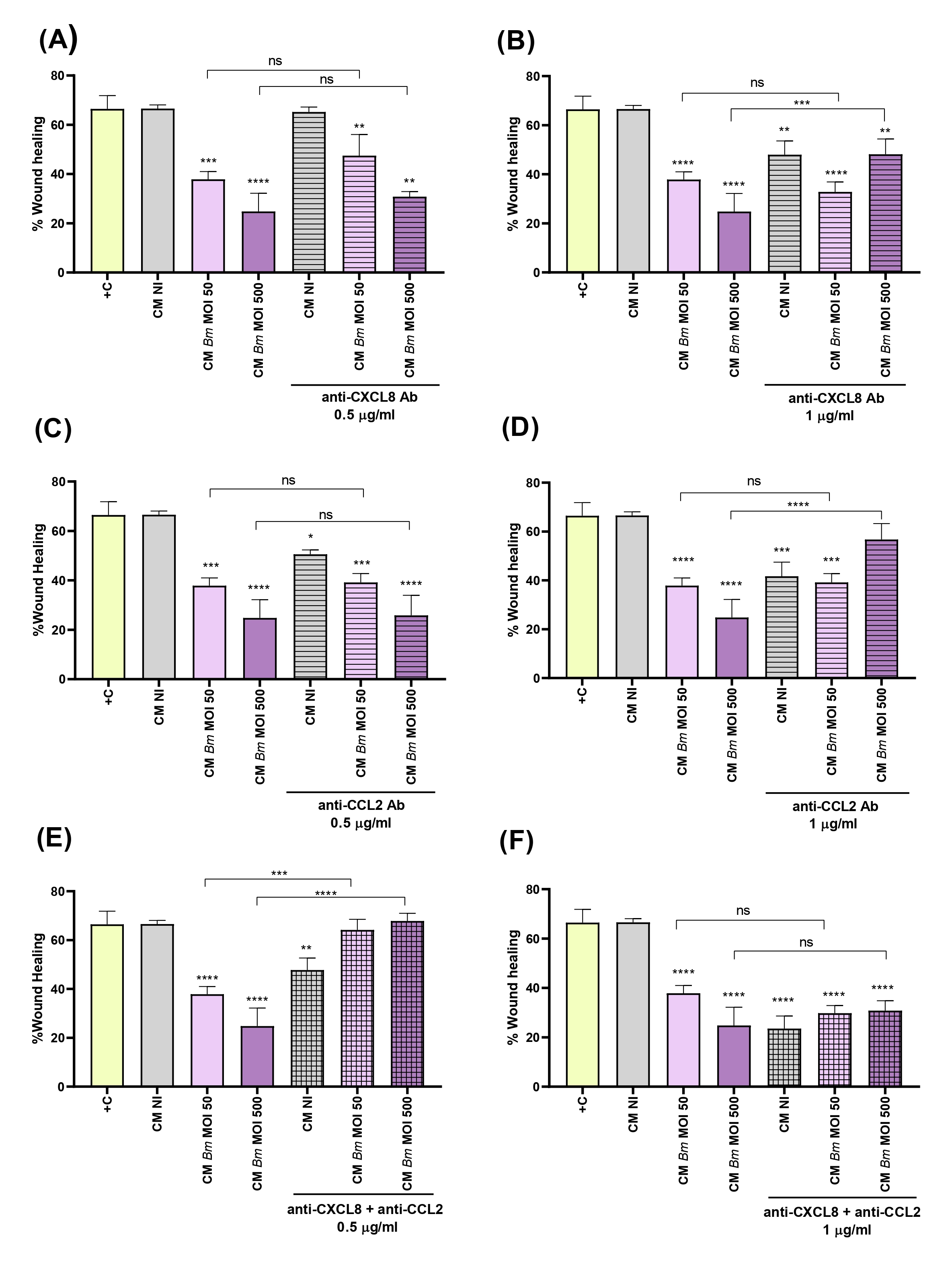

Supplement: Supplementary file 1 [file ijms-26-08189-s001.zip › Figure S3.jpg]
